# Longitudinal experiences and risk factors for common mental health problems and suicidal behaviours among female sex workers in Nairobi, Kenya

**DOI:** 10.1017/gmh.2022.44

**Published:** 2022-08-18

**Authors:** Alicja Beksinska, Pooja Shah, Mary Kungu, Rhoda Kabuti, Hellen Babu, Zaina Jama, Mamtuti Panneh, Emily Nyariki, Chrispo Nyabuto, Monica Okumu, Pauline Ngurukiri, Erastus Irungu, Rupert Kaul, Janet Seeley, Mitzy Gafos, Tara S. Beattie, Helen A. Weiss, Joshua Kimani

**Affiliations:** 1 Department of Global Health and Development, Faculty of Public Health and Policy, London School of Hygiene and Tropical Medicine, London, UK; 2 University of Nairobi College of Health Sciences, Institute of Tropical and Infectious Diseases, UK Partners for Health and Development in Africa (PHDA), Nairobi, Kenya; 3 Department of Medicine, University of Toronto, Toronto, Canada; 4 London School of Hygiene and Tropical Medicine, Faculty of Epidemiology and Public Health, Department of Infectious Disease Epidemiology, MRC International Statistics & Epidemiology Group, London, UK

**Keywords:** Female sex workers, mental health

## Abstract

**Background:**

Female sex workers (FSWs) are at high risk of mental health problems and suicide risk. Few longitudinal studies have examined risk factors for poor mental health among FSWs.

**Methods:**

Maisha Fiti is a longitudinal study among FSWs randomly selected from Sex Worker Outreach Programme clinics across Nairobi. Behavioural-biological survey data were collected at baseline (*n* = 1003, June–December 2019), midline (*n* = 366) (Jan–March 2020) and endline (*n* = 877) (June 2020–Jan 2021). Women reporting mental health problems were offered counselling services. Multivariable mixed logistic regression models were used to examine factors associated with mental health problems and suicidal behaviours.

**Results:**

There was a decline in the proportion of women reporting any mental health problem (depression and/or anxiety and/or PTSD) (baseline: 29.9%, midline: 13.3%, endline: 11.8%). There was strong evidence that any mental health problem was associated with recent hunger (aOR 1.99; 95% CI 1.37–2.88) and recent violence from non-intimate partners (2.23; 95% CI 1.55–3.19). Recent suicidal behaviour prevalence was similar across survey rounds (baseline: 10.2%; midline: 10.2%; endline: 10.4%), and was associated with recent violence from non-intimate partners (aOR 1.96; 95% CI 1.31–2.95), recent hunger (aOR 1.69; 95% CI 1.15–2.47) and having an additional employment to sex work (aOR 1.50; 95% CI 1.00–2.23).

**Conclusions:**

Our study found a decline in mental health problems but high levels of persistent suicidal behaviours among FSWs. Syndemic risk factors including food insecurity and violence were longitudinally associated with mental health problems and recent suicidal behaviours. There is a need for accessible mental health services for FSWs, alongside structural interventions addressing poverty and violence.

## Introduction

Mental health problems are a major contributor to the global burden of disease (Murray *et al*., 2020). There is a high burden of untreated common mental health problems with a treatment gap greater than 90% in low-income countries (Thornicroft *et al*., 2017). Women in low and middle-income countries (LMICs) are at greater risk than men according to the 2019 Global Burden of Disease study, with a higher prevalence of depression (4.5%; 95% CI 4.0–5.0 *v*. men: 3.0%; 95% CI 2.7–3.4) and anxiety disorders (4.3%; 95% CI 3.6–5.2% *v*. men: 2.8%; 95% CI 2.3–3.3%) (Murray *et al*., 2020). Risk factors for mental health problems include demographic and socio-economic factors such as age, gender, poverty and educational status, exposure to violence and harmful alcohol and substance use (Patel *et al*., 2016). Untreated mental health problems result in short- and long-term impacts on quality of life and increase the risk of mortality from suicide (Thornicroft *et al*., 2017).

Sex work [defined as the receipt of money or goods in exchange for sexual services (UNAIDS, 2002)] is criminalised in most parts of the world (Platt *et al*., 2018). Female sex workers (FSWs) face intersecting health and social risks, such as poverty, gender inequality, police arrest, harmful alcohol and substance use, violence from clients and intimate partners and a high prevalence of HIV and other sexually transmitted infections (STIs) (Shannon *et al*., 2015; Platt *et al*., 2018). These overlapping risk factors which often perpetuate each other are known as syndemic factors (Singer and Clair, 2003; Buttram *et al*., 2014). A recent systematic review and meta-analysis examining mental health problems among FSWs in LMICs found high levels of mental health problems including depression, anxiety and PTSD, compared to the general population (Beattie *et al*., 2020). The authors found strong evidence of associations between depression and recent violence experience (OR 2.3; 95% CI 1.3–4.2), alcohol use (OR 2.1; 95% CI 1.4–3.2), inconsistent condom use with clients (OR 1.6; 95% CI 1.2–2.1) and HIV (OR 1.4; 95% CI 1.1–1.8) (Beattie *et al*., 2020) as well as associations between recent suicide attempt with ever experiencing violence (OR 3.5; 95% CI 2.2–5.5), alcohol use (OR 1.6; 1.0–2.5) and HIV (OR 1.4; 95% CI 1.1–1.8).

Currently, there is an evidence gap both in (1) longitudinal studies examining the burden of mental health problems among FSWs over time, and (2) studies assessing the effect of interventions addressing mental health and associated risk factors such as violence among FSWs. The majority of research among FSWs has focussed on addressing HIV/STI risk through behavioural and biomedical interventions (Platt *et al*., 2018), although more recently some interventions have targeted key structural drivers such as violence (Beattie *et al*., 2015). Beattie *et al*. systematic review on mental health among FSWs reported both a lack of longitudinal and intervention studies among FSWs (Beattie *et al*., 2020). Longitudinal studies are crucial to understand pathways of causation between potential risk factors and mental health problems to identify targets for upstream interventions. In addition, there are limitations in interpreting the current evidence due to the varied use of measurement tools and cut-off scores (Beattie *et al*., 2020) to measure mental health problems and associated risk factors.

This paper aims to address this evidence gap by examining temporal trends in mental health problems and longitudinal relationships with risk factors such as violence, poverty and alcohol/substance use using data from the Maisha Fiti Study. Maisha Fiti is a mixed-methods longitudinal study among FSWs in Nairobi, Kenya which aims to examine associations between violence experience, mental health problems, harmful alcohol and substance use as well as biological changes to the immune system such as genital tract inflammation and HIV among FSWs. Previously reported findings from Maisha Fiti indicate a high baseline prevalence of depression, anxiety, PTSD and suicidal behaviours, measured using validated mental health tools, which were associated with syndemic risk factors including recent violence, poverty and harmful alcohol/substance use (Beksinska *et al*., 2021). Women were offered psychological counselling during the study and we report on the uptake and assess the effects of a brief psychological counselling service for FSWs. Our findings will be used to design and deliver much needed holistic mental health interventions tailored for FSWs.

## Methods

### Study design and sampling

**Fig. 1.**
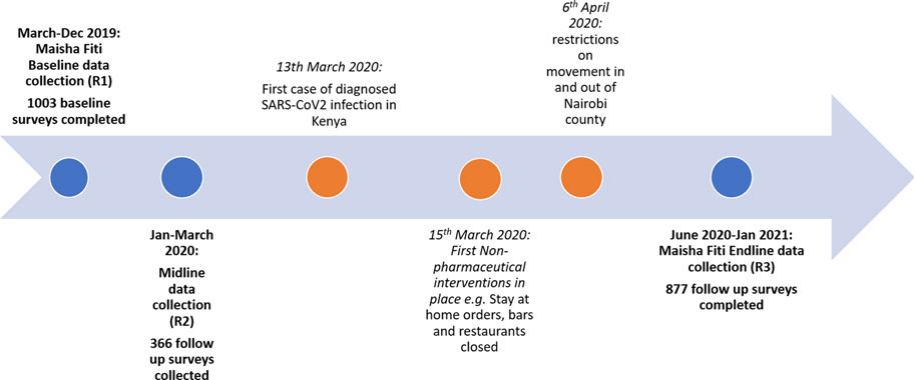
Timeline of baseline and follow-up surveys in relation to COVID-19 and prevention measures in Kenya.

The Maisha Fiti study was designed in consultation with FSWs in Nairobi and with community mobilisers and staff working at seven SWOP clinics. These clinics are delivered with the support of Partners for Health and Development Africa (PHDA) to provide HIV/STI prevention and treatment services to FSWs across Nairobi. Women were randomly selected by the study team from the seven SWOP clinic attendance lists of all clinic attendees in the previous 12 months, and invited to enrol in the study. Sample size calculations and sampling methodology have been described previously (Beksinska *et al*., 2021). Initially follow-up data collection was planned one year after baseline and began in January 2020. Due to the onset of the COVID-19 epidemic in Kenya, data collection was paused in March 2020, resumed in June 2020 and was completed in January 2021. At the time, it was decided to re-survey all women (*n* = 877) due to the delay and the likely change in women's mental health experiences as a result of COVID-19. Data collected January–March 2020 (*n* = 366) were considered as midline data (Fig. 1).

Eligibility criteria were women aged 18–45 years, who self-identified as sex workers (exchanged money for sex or accepted goods as payment), had attended one of the SWOP clinics in the past 12 months, were not pregnant or breast-feeding and did not have an underlying chronic illness (other than HIV) that was likely to alter host immunology. Selected women were telephoned, given information about the study and the opportunity to ask any questions and invited to attend the study clinic. Interested women were screened for eligibility and received detailed information about the study verbally and via the participant information leaflet (in English or Swahili). For women with low literacy, this information was read to them by study staff. Consenting participants completed a behavioural-biological survey. Those found to have mental health or alcohol/substance use problems were referred to a trained psychological counsellor based in the study clinic. All women who tested positive for HIV at any of the three study visits were counselled and encouraged to enrol in HIV care. All women who tested positive for STIs were offered appropriate treatment free of charge.

### Behavioural-biological survey

Time-invariant measures collected at baseline include age, marital status, number of children, religion, literacy, socio-economic status (SES). Time-variant measures including mental health problems, recent physical and/or sexual violence experience, alcohol/substance use, sexual risk behaviours and recent indicators of financial stress (e.g. missed a meal in the past week due to financial difficulties) were collected at baseline, midline and endline. Validated tools with high reliability and validity [see baseline paper for more details (Beksinska *et al*., 2021)] were used for assessing depression – Patient Health Questionnaire (PHQ-9) (score ⩾15 = moderate/severe depressive disorder) (Kroenke *et al*., 2001); anxiety – Generalised Anxiety Disorder (GAD-7) (score ⩾10 = moderate/severe anxiety) (Spitzer *et al*., 2006) and PTSD – Harvard Trauma Questionnaire (HTQ-17) (Kleijn *et al*., 2001). Suicide risk was measured by a two-item questionnaire which included recent suicidal ideation (‘*having thoughts about ending your life in the last 30 days*’) and recent suicide attempt (‘*having attempted to end your life in the last 30 days*’). Due to the small number of women reporting a recent suicide attempt, and the significant overlap in women reporting a recent suicide attempt and recent suicidal ideation, we combined these measures into a dichotomous variable including women who reported recent suicidal thoughts and/or recent suicide attempt under the term ‘recent suicidal behaviours’. The term ‘suicidal behaviours’ encompasses a continuum of risk from suicidal thoughts to planning to attempts and is a recognised definition in the psychiatric literature (Posner *et al*., 2007; Nock *et al*., 2008; O'Connor and Nock, 2014).

The WHO ASSIST (Alcohol, Smoking and Substance Involvement Screening Test) tool was used to measure harmful alcohol use (cut-off scores: moderate risk >11; high risk >27) and other substance use (cut-off scores: moderate risk >4; high risk >27) in the last 3 months including amphetamines, cannabis, cocaine, hallucinogens, sedatives and inhalants [World Health Organisation (WHO), 2010].

The WHO Violence Against Women 13-item questionnaire which measures frequency and severity of intimate partner violence (IPV) was adapted to also include violence perpetrated by non-IPs (e.g. police, strangers, clients, etc.) in the past 6 months (World Health Organisation, 2005).

Women were offered psychological counselling with a trained psychological counsellor if they reported moderate/severe depression, recent suicidal ideation/self-harm, recent violence or moderate/high risk alcohol use. As part of the study, training in an evidence-based brief psychological intervention for depression [Healthy Activity Program (HAP)] (Patel *et al*., 2017) and alcohol use disorders [Counselling for Alcohol Problems (CAP)] (Nadkarni *et al*., 2017) was delivered to lay counsellors (HIV testing and screening counsellors) and peer sex workers, working at existing SWOP clinics. Uptake of Maisha Fiti study counselling at baseline was self-reported at midline and endline.

### Laboratory methods

Biological samples were collected at baseline, midline and endline. Urine samples were collected to test for pregnancy, *Neisseria gonorrhoeae* and *Chlamydia trachomatis*. Blood was collected for *Treponema pallidum* (syphilis) testing and HIV status. Self-collected vaginal swabs tested for *Trichomonas vaginalis* and bacterial vaginosis. Laboratory methods have been previously described in detail (Beksinska *et al*., 2021).

### Conceptual framework

**Fig. 2.**
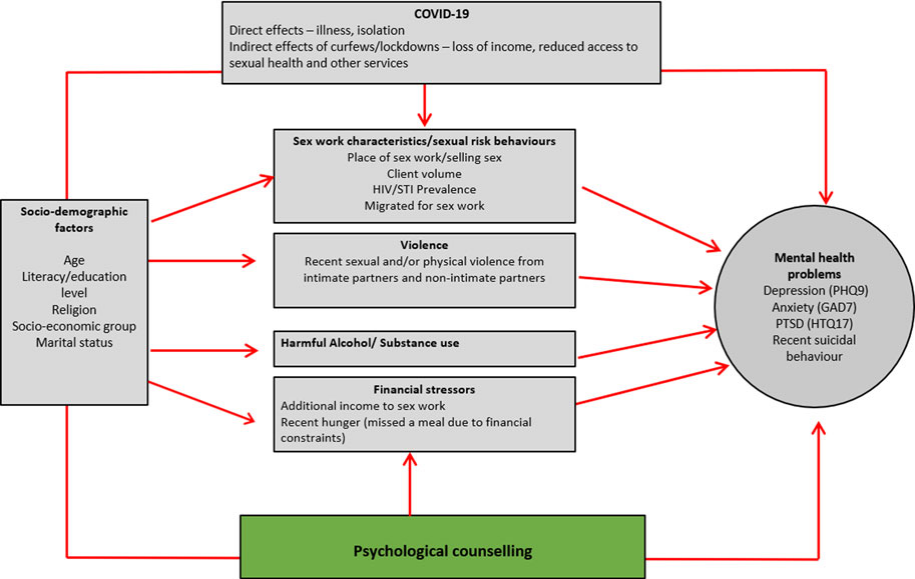
Conceptual framework exploring potential correlates of mental health problems and suicidal behaviours over time.

We developed a conceptual framework (Fig. 2) to explore correlates of mental health problems and recent suicidal behaviours using an eco-social life course theory approach (Krieger, 2012). This is based on the framework in our baseline analysis (Beksinska *et al*., 2021) and adapted to consider longitudinal associations, the impact of counselling and the potential effects of COVID-19 on mental health outcomes.

### Statistical analyses

Data were double-entered and statistical analyses were conducted in STATA 16.1 (Stata Inc., College Station, TX, USA). As women aged <25 years old were over-sampled, analyses included weighting for age group using inverse probability weights. Key baseline socio-demographic characteristics were compared between those individuals participating at both baseline and endline (*n* = 877), with those seen at baseline only (*n* = 126) using Pearson's *χ*^2^ tests. Data from baseline, midline and endline surveys were used to describe changes in the prevalence of mental health problems and recent suicidal behaviours over time. Changes in mental health problems between survey rounds were calculated using mixed-effects logistic regression to adjust for repeated measures over time.

The two outcomes of interest were (1) any recent mental health problem (moderate/severe depression *and/or* moderate/severe anxiety *and/or* PTSD) and (2) suicidal behaviour (reported suicidal attempts *and/or* ideation). Analyses for each outcome were conducted using repeated measures mixed-effects logistic regression to allow for individual-level clustering, and adjusting for clinic-level clustering by including SWOP clinic as a fixed effect. Associations were estimated using odds ratios (OR), with *p* values obtained using the adjusted Wald test due to data being weighted. In this repeated measures model, the ORs are assumed constant for the association of outcomes at midline with exposures at baseline, and for outcomes at endline with exposures at midline. Initial ORs were estimated as crude, and a minimally-adjusted model which included the set of *a priori* defined potential confounders (age group at enrolment, religion, literacy, SES and marital status). Variables which were either associated with the outcome in the minimally-adjusted model (*p* < 0.10), or which were *a priori* defined potential confounders, were included in a multivariable model and were retained in the final model if they were independently associated with the outcome (*p* < 0.10). Uptake of counselling prior to endline was identified as an *a priori* effect-modifier to test the hypothesis that counselling could modify the association between baseline risk factors and endline mental health problems. Variables in the final multivariable model were assessed for effect-modification with uptake of counselling.

## Results

### Study population and sex work characteristics

Of the 1039 eligible FSWs, 1003 (96.5%) women participated in baseline surveys. Of these, 366 (36.5%) participated at midline and 877 (87.4%) at endline. Of the women that participated at midline 94.0% took part at endline. At baseline, the median age of participants was 32 years (range 18–45; IQR 26–39) and most were currently or previously married (81.2%). The majority of women reported their religion to be Protestant (54.4%) or Catholic (36.9%). The mean age at first sex was 16 years (IQR 15–18; s.d. 2.6). Almost half (43.7% at baseline, 45.7% at endline) of women reported additional employment to sex work, and there was an increase in the proportion of women reporting recent hunger due to financial difficulties during follow-up (33.0% at baseline; 54.3% at endline). Most women worked from a lodge/hotel/rented room (91.4% at baseline, 85.2% at endline). The median client volume per week (reported clients in last 7 days) declined from 3 at baseline to 2 at endline. HIV prevalence was 28.0% at baseline, with no new HIV diagnoses among those followed up. There was no change in the proportion of women reporting condom use at last sex (77.1% at baseline; 77.5% at endline). Reported harmful alcohol use declined between survey rounds (29.8% at baseline; 13.4% at endline), as did other harmful substance use (30.7% at baseline; 24.3% at endline), and reported sexual or physical violence from non-IPs (55.0% at baseline; 20.9% at endline) and from intimate partners (30.9% at baseline; 12.1% at endline).

Factors associated with loss-to-follow-up by endline were HIV status (higher follow-up among women living with HIV; *p* = 0.001) and reported recent violence from non-IPs (higher follow-up among those not reporting violence; *p* = 0.05) (online Supplementary Appendix A).

### Temporal changes in mental health problems

**Fig. 3.**
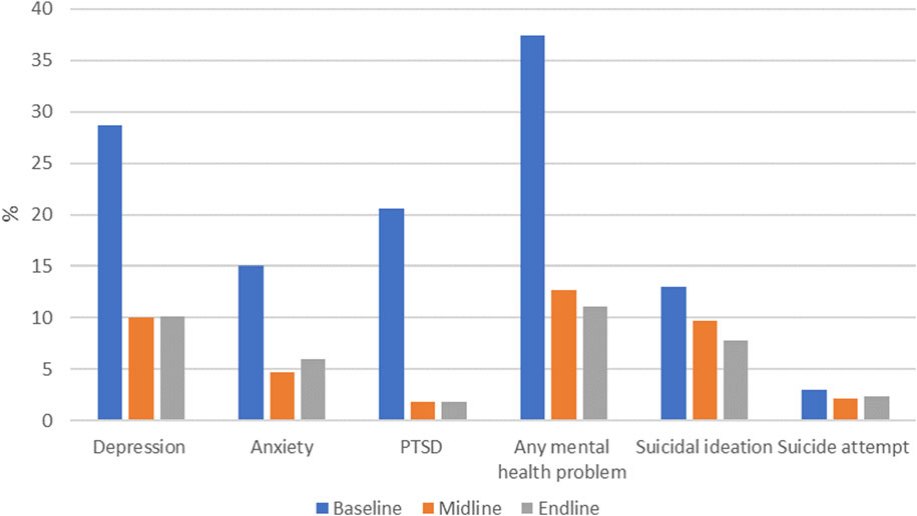
Temporal changes in prevalence of mental health problems among FSWs in Nairobi who completed baseline, midline and endline surveys (*n* = 366).

The prevalence of any mental health problem among participants declined over time (Table 1) from 29.9% at baseline to 13.3% at midline and 11.8% at endline (*p* value for trend <0.0001). The decline was seen for depression, anxiety and PTSD, respectively (Table 1). The prevalence of recent suicidal behaviour did not change over time (10.2% at baseline; 10.2% at midline; 10.4% at endline) (Table 1). Findings were similar among the 366 women who completed baseline, midline and endline surveys (Fig. 3).

**Table 1.** Temporal changes in prevalence of mental health problems among FSWs at baseline, midline and endline surveys

	Baseline		Midline		Endline		*p* value^a^
	(*n* = 1003)	% (95% CI)	(*n* = 366)	% (95% CI)	(*n* = 877)	% (95% CI)	<0.0001
Any mental health problem (depression/anxiety/PTSD)	293	29.9 (27.2–32.8)	47	13.3 (10.2–17.3)	97	11.8 (9.9–14.2)	<0.0001
Depression	222	23.2 (20.7–25.9)	40	11.6 (8.6–15.4)	87	10.6 (8.7 −12.9)	<0.0001
Anxiety	109	11.0 (9.3–13.1)	17	4.8 (3.0–7.5)	39	4.8 (3.5–6.4)	<0.0001
PTSD	138	14.2 (12.2–16.5)	7	1.8 (0.8–3.8)	16	1.9 (1.1–3.0)	<0.0001
Suicidal ideation (last 6 months)	99	10.0 (8.3–12.0)	36	10.2 (7.5–13.9)	90	10.4 (8.5–12.6)	0.9
Suicide attempt (last 6 months)	27	2.6 (1.8–3.8)	7	1.9 (0.9–4.0)	21	2.4 (1.5–3.6)	0.7
Suicidal behaviour (recent suicidal ideation and/or attempt)	101	10.2 (8.5–12.2)	36	10.2 (7.5–13.9)	91	10.4 (8.4–12.6)	0.9

a Mixed-effects logistic regression.

We examined persistent and incident mental health problems among the 877 women who participated at baseline and endline (Table 2). Among 253 women who reported any mental health problems at baseline, 69 (27.3%) had a persistent mental health problem, and among 624 women without any mental health problems at baseline, 28 (4.5%) reported an incident mental health problem (Table 2). Approximately one-third of women with each condition at baseline had persistence of that condition at endline (depression: 31.7%, anxiety 29.8%, PTSD 29.8%). Co-morbid persistence of mental health problems was common. Among women with persistent depression nearly half (40.2%) reported persistent anxiety compared to women without persistent depression (4.1%) and 13.3% reported persistent PTSD (*v*. 0.8% amongst those without depression). The incidence of new mental health problems was low for each condition with 3.9% of women reporting incident depression, 1.4% anxiety and 0.5% PTSD.

**Table 2.** Persistent and incident mental health problems/suicidal behaviours between baseline and endline surveys (*n* = 877)

	Persistent^a^ *n* (%)	Incident^b^ *n* (%)
Any MH problem (depression/anxiety/PTSD)	69/253 (27.3%)	28/624 (4.5%)
Depression	60/189 (31.7%)	27/685 (3.9%)
Anxiety	28/94 (29.8%)	11/783 (1.4%)
PTSD	12/118 (10.2%)	4/751 (0.5%)
Suicidal ideation	57/85 (67.1%)	33/792 (4.2%)
Suicide attempt	14/21 (60%)	7/856 (0.8%)
Suicidal behaviour	57/86 (66.3%)	34/791 (4.3%)

a Persistent, mental health problem at baseline and endline.

b Incident, new mental health problem at endline.

Of the women with suicidal behaviour reported at baseline, two-thirds (66.3%) reported these at endline. Among women with a persistent mental health problem, one-third (33.6%) had persistent suicidal behaviours (*v*. 4.0% among women without persistent mental health problems).

### Temporal associations between baseline exposures and mid/endline mental health and suicidal behaviour outcomes

To explore time-dependent associations, we assessed outcomes at midline and endline with exposures at baseline and midline respectively, adjusting for within-person correlation. The variables independently associated with any mental health problem or suicidal behaviours after adjusting for *a priori* confounders (Tables 3 and 4) were retained in the final multivariable model. In the final adjusted models, variables associated with increased odds of mental health problems include older age (age 25–34: aOR 2.68; 95% CI 1.40–5.12; age >35: aOR 2.29; 95% CI 1.19–4.42; *p* = 0.01), Muslim *v*. Catholic religion (aOR 2.74; 95% CI 1.43–5.24; *p* < 0.001), ever being married (aOR 2.07; 95% CI 1.20–3.56; *p* = 0.009), recent hunger (aOR 1.99; 95% CI 1.37–2.88; *p* < 0.001) and recent violence from non-IPs (2.23; 95% CI 1.55–3.19; *p* < 0.0001) (Table 5). There was some evidence that women with a higher client volume (>10 clients per week *v*. <5 clients) had lower odds of mental health problems (aOR 0.45; 95% CI 0.20–1.00; *p* 0.07). HIV/STIs were not associated with any mental health problems in univariate and adjusted analysis. Overall, 42.9% (*n* = 372) of women reported attending any Maisha Fiti counselling at mid/endline. There was no evidence that attending counselling modified the effect of any associations with mental health problems (Table 5).

**Table 3.** Longitudinal associations between any mental health problems (moderate/severe depression and/or anxiety and /or PTSD) and key exposures including socio-demographic variables at baseline, financial stressors, sex work characteristics, recent violence experience and alcohol/substance

		% with mental health problems at baseline (*n* = 293/1003)	% with mental health problems at midline (*n* = 47/366)	% with mental health problems at endline (*n* = 97/877)	Crude OR (95% CI)	*p* value	Minimally adjusted OR^a^	*p* value
Age (years)	<25	9.2	4.4	4.3	1.0		1.0	
	25–34	37.0	35.6	46.3	3.05 (1.62–5.74)		2.68 (1.40–5.12)	
	35+	53.8	60.0	49.5	2.73 (1.47–5.08)	0.002	2.29 (1.19–4.42)	0.01
Literacy	Illiterate	19.6	17.8	25.3	1.0		1.0	
	Literate	80.4	82.2	74.7	0.68 (0.45–1.03)	0.07	0.85 (0.55–1.31)	0.5
Religion	Catholic	35.3	44.4	34.4	1.0		1.0	
	Protestant	54.2	43.4	46.3	0.81 (0.56–1.17)		0.79 (0.54–1.15)	
	Muslim	5.6	10.0	12.4	2.83 (1.54–5.20)		2.74 (1.43–5.24)	
	Other/none	4.9	2.2	7.0	1.69 (0.77–3.72)	<0.001	1.83 (0.83–4.00)	<0.001
Socio-economic status (SES)	Low/low-middle	42.2	52.2	42.5	1.0		1.0	
	Middle	21.8	22.2	20.4	0.88 (0.56–1.37)		0.87 (0.55–1.38)	
	Upper middle/upper	36.0	25.6	37.1	0.68 (0.47–0.99)	0.1	0.67 (0.44–1.04)	0.2
Ever married	No	15.8	6.7	11.3	1.0		1.0	
	Yes	84.2	93.3	88.7	2.33 (1.36–4.00)	0.002	2.07 (1.20–3.56)	0.009
Additional employment to sex work	No	48.9	55.6	43.5	1.0		1.0	
	Yes	51.1	44.4	56.5	0.86 (0.61–1.20)	0.4	0.84 (0.59–1.21)	0.4
Recent hunger (missed a meal in the last 7 days due to financial constraints)	No	52.6	50.0	26.9	1.0		1.0	
	Yes	47.4	50.0	73.1	2.41 (1.72–3.38)	<0.0001	2.19 (1.52–3.16)	<0.0001
Place of sex work	Lodge/hotel/rented room	89.3	91.1	88.3	1.0		1.0	
	Other public place	3.4	4.5	2.13	1.86 (0.78–4.43)		1.63 (0.67–3.96)	
	Home	7.3	4.4	9.6	1.42 (0.72–2.78)	0.2	1.39 (0.67–2.88)	0.4
Client volume/week	<5	63.2	84.1	82.5	1.0		1.0	
	5–9	21.1	11.4	13.4	0.78 (0.52–1.18)		0.77 (0.51–1.18)	
	10+	15.7	4.6	4.2	0.50 (0.27–0.94)	0.07	0.48 (0.25–0.92)	0.06
Condom use last sex	No	26.7	37.8	25.3	1.0		1.0	
	Yes	73.3	62.2	74.7	0.77 (0.53–1.13)	0.2	0.83 (0.56–1.23)	0.3
Any STI at baseline	No	87.0	95.6	86.8	1.0		1.0	
	Yes	13.0	4.5	13.2	1.22 (0.72–2.05)	0.5	1.16 (0.68–1.99)	0.6
HIV status	Negative	73.1	60.0	71.0	1.0		1.0	
	Positive	26.9	40.0	29.0	1.16 (0.81–1.66)	0.8	1.06 (0.72–1.57)	0.8
Migrated for sex work (sex work outside Nairobi last 6 months)	No	72.7	65.4	59.7	1.0		1.0	
	Yes	27.4	34.6	40.3	1.09 (0.74–1.61)	0.7	1.03 (0.69–1.54)	0.9
Any recent sexual and/or physical non-IP violence	No	32.8	54.5	59.2	1.0		1.0	
	Yes	67.2	45.5	40.8	2.26 (1.59–3.21)	<0.0001	2.23 (1.56–3.18)	<0.0001
Any recent sexual and/or physical IP violence	No	60.6	67.8	80.1	1.0		1.0	
	Yes	39.4	32.2	19.9	1.29 (0.90–1.85)	0.2	1.21 (0.83–1.78)	0.3
Recent arrest	No	63.1	75.6	81.7	1.0		1.0	
	Yes	36.9	24.4	18.3	1.18 (0.82–1.69)	0.4	1.08 (0.74–1.58)	0.7
Alcohol use problem (ASSIST moderate/high)	No	49.7	54.6	65.9	1.0		1.0	
	Yes	50.3	45.5	34.1	1.15 (0.80–1.67)	0.4	1.12 (0.76–1.65)	0.6
Substance use problem (ASSIST moderate/high)	No	56.6	73.4	62.4	1.0		1.0	
	yes	43.5	26.7	37.6	1.26 (0.89–1.79)	0.2	1.16 (0.79–1.70)	0.5

a Adjusted for *a priori* defined potential confounders (age at baseline, literacy, religion, SES, ever married) and SWOP clinic.

**Table 4.** Longitudinal associations between reported suicidal behaviour (recent suicide attempt and/or ideation) and key exposures including socio-demographic variables at baseline, financial stressors, sex work characteristics, recent violence experience and alcohol/substance

		% suicidal behaviour at baseline (*n* = 101/1003)	% suicidal behaviour at midline (*n* = 36/366)	% suicidal behaviour at endline (*n* = 91/877)	Crude OR (95% CI)	*p* value^a^	Minimally adjusted OR	*p* value^a^
Age (years)	<25	10.3	4.3	10.9	1.0		1.0	
	25–34	41.6	46.4	44.0	1.45 (0.86–2.44)		1.26 (0.74–2.16)	
	35+	48.1	49.3	45.2	1.07 (0.63–1.79)	0.2	0.89 (0.51–1.55)	0.2
Literacy	Illiterate	27.4	20.3	26.9	1.0		1.0	
	Literate	72.7	79.7	73.1	0.60 (0.39–0.92)	0.02	0.62 (0.39–0.99)	0.04
Religion	Catholic	36.1	31.9	34.7	1.0		1.0	
	Protestant	48.6	59.4	54.9	1.17 (0.79–1.71)		1.25 (0.84–1.85)	
	Muslim	8.7	2.9	6.7	1.27 (0.56–1.71)		1.32 (0.56–3.09)	
	Other/none	6.6	5.8	3.7	1.42 (0.57–3.50)	0.8	1.45 (0.56–3.78)	0.7
Socio-economic status (SES)	Low/low-middle	56.4	56.5	44.5	1.0		1.0	
	Middle	19.9	34.8	29.9	1.32 (0.87–2.01)		1.52 (0.98–2.35)	
	Upper middle/upper	23.8	8.7	25.6	0.39 (0.24–0.62)	<0.0001	0.41 (0.25–0.68)	<0.0001
Ever married	No	16.4	5.8	11.6	1.0		1.0	
	Yes	83.6	94.2	88.4	2.28 (1.29–4.02)	0.005	2.31 (1.29–4.13)	0.005
Additional employment to sex work	No	48.1	52.2	50.0			1.0	
	Yes	51.9	47.8	50.0	1.27 (0.88–1.82)	0.2	1.42 (0.98–2.07)	0.06
Recent hunger (missed a meal in the last 7 days due to financial constraints)	No	43.4	33.3	21.4			1.0	
	Yes	56.6	66.7	78.7	2.36 (1.65–3.38)	<0.0001	1.86 (1.29–2.68)	<0.001
Place of sex work	Lodge/hotel/rented room	86.2	94.2	89.0	1.0		1.0	
	Other public place	6.1	0.0	4.3	1.54 (0.60–3.98)		1.40 (0.51–3.84)	
	Home	7.7	5.8	6.7	1.07 (0.50–2.29)	0.7	1.08 (0.50–2.32)	0.8
Client volume/week	<5	59.2	66.7	60.4	1.0		1.0	
	5–9	27.9	18.8	25.6	1.23 (0.82–1.85)		1.19 (0.78–1.81)	
	10+	12.9	14.5	14.0	0.86 (0.47–1.54)	0.5	0.88 (0.47–1.64)	0.6
Condom use last sex	No	22.2	26.1	32.9				
	Yes	77.8	73.9	67.1	0.73 (0.49–1.09)	0.1	0.78 (0.52–1.22)	0.3
Any STI at baseline	No	86.9	97.1	88.7			1.0	
	Yes	13.1	2.9	11.3	1.11 (0.63–1.96)	0.7	0.99 (0.55–1.80)	1.0
HIV status	Negative	72.1	52.2	70.7			1.0	
	Positive	27.9	47.8	29.3	1.29 (0.88–1.89)	0.2	1.20 (0.79–1.81)	0.4
Migrated for sex work Sex work outside Nairobi last 6 months	No	70.7	65.3	58.7			1.0	
	Yes	29.3	34.7	41.3	1.03 (0.68–1.55)	0.9	1.08 (0.70–1.67)	0.7
Any recent sexual and/or physical non-IP violence	No	23.5	31.9	53.7			1.0	
	Yes	76.5	68.1	46.3	2.28 (1.57–3.32)	<0.0001	2.03 (1.37–3.00)	<0.0001
Any recent sexual and/or physical IP violence	No	55.8	66.7	86.6			1.0	
	Yes	44.3	33.3	13.4	2.28 (1.57–3.32)	<0.0001	1.37 (0.92–2.04)	0.1
Recent arrest	No	52.4	63.8	76.8			1.0	
	Yes	47.6	36.2	23.2	1.64 (1.13–2.38)	0.009	1.49 (1.01–2.20)	0.05
Alcohol use problem (ASSIST moderate/high)	No	48.4	69.7	78.2			1.0	
	Yes	51.6	30.3	21.8	1.28 (0.85–1.94)	0.3	1.28 (0.85–1.94)	0.2
Substance use problem (ASSIST moderate/high)	No	49.7	65.2	71.4			1.0	
	Yes	50.3	34.8	28.6	1.12 (0.74–1.70)	0.2	1.16 (0.79–1.69)	0.5

a Adjusted Wald test.

**Table 5.** Multivariable analysis to examine longitudinal associations with mental health outcomes and effect-modification with uptake of counselling

		Final adjusted OR (95% CI)	*p* value^a^	Did not attend counselling	Attended counselling	*p* value^a^
*Baseline demographics*^b^						
Age (years) at baseline^b^	<25	1.0		1.0	1.0	
	25–34	2.68 (1.40–5.12)		2.13 (0.72–6.24)	3.49 (1.19–10.2)	
	35+	2.29 (1.19–4.42)	0.01	1.46 (0.51–4.21	3.84 (1.33–11.06	0.2
Religion	Catholic	1.0		1.0	1.0	
	Protestant	0.79 (0.54–1.15)		0.81 (0.47–1.40)	0.69 (0.40–1.19	
	Muslim	2.74 (1.43–5.24)		3.22 (1.13–9.17)	1.55 (0.54–4.41)	
	Other/none	1.83 (0.83–4.00)	<0.001	1.35 (0.48–3.84	2.70 (0.95–7.68)	0.5
Ever married	Yes	2.07 (1.20–3.56)	0.009	2.01 (0.84–4.79)	2.65 (1.11–6.31)	0.6
*Financial stressors, sex work characteristics, sexual risk behaviours, violence and alcohol/substance use (time variant exposures)*^c^						
Recent hunger – missed a meal in the last 7 days	Yes	1.99 (1.37–2.88)	<0.001	1.77 (1.03–3.03)	2.23 (1.40–3.82)	0.5
Clients per week	<5	1.0		1.0	1.0	
	5–9	0.68 (0.40–1.16)		0.63 (0.28–1.44)	0.76 (0.34–1.73)	
	10+	0.45 (0.20–1.00)	0.07	0.23 (0.09–0.8)	0.78 (0.27–2.25)	0.4
Any recent sexual and/or physical non-IP violence	Yes	2.23 (1.55–3.19)	<0.0001	2.27 (1.36–3.80)	2.26 (1.35–3.79)	1.0

a Adjusted Wald test.

b Adjusted for all *a priori* potential confounders (age at baseline, religion, SES, literacy, ever married) and SWOP clinic.

c Adjusted for all *a priori* potential confounders (age at baseline, religion, SES, literacy, ever married), clinic, violence from non-IPs, hunger and clients per week.

In adjusted analysis women with higher literacy (aOR 0.62; 95% CI 0.39–0.99) and higher SES (middle upper/upper *v*. lower middle/low aOR 0.41; 95% CI 0.25–0.68) had reduced odds of suicidal behaviour (Table 6). There was an increased odds of suicidal behaviour among women who had ever been married (aOR 2.31; 95% CI 1.29–4.13), reported an additional employment to sex work (aOR 1.50; 95% CI 1.00–2.23), experienced recent hunger (aOR 1.69; 95% CI 1.15–2.47) and among those who reported recent violence from non-IPs (aOR 1.96; 95% CI 1.31–2.95).

**Table 6.** Multivariable analysis to examine longitudinal associations between exposures and suicidal behaviours (suicidal ideation and/or attempt) and effect-modification with uptake of counselling

		Adjusted OR (95% CI)	*p* value^a^	Did not attend counselling	Attended counselling	*p* value^a^
*Baseline demographics*^b^						
Literacy	Illiterate	1.0		1.0	1.0	
	Literate	0.62 (0.39–0.99)	0.04	1.14 (0.61–2.13)	0.35 (0.18–0.65)	0.01
Socio-economic status (SES)	Low/low-middle	1.0		1.0	1.0	
	Middle	1.52 (0.98–2.35)		2.43 (1.25–4.71)	0.88 (0.45–1.70)	
	Upper middle/upper	0.41 (0.25–0.68)	<0.0001	0.54 (0.26–1.07)	0.33 (0.16–0.66)	0.07
Ever married	No	1.0		1.0	1.0	
	Yes	2.31 (1.29–4.13)	0.005	2.05 (0.76–5.53)	2.94 (1.09–7.95)	0.6
*Financial stressors, sex work characteristics, sexual risk behaviours, violence and alcohol/substance use (time variant exposures)*^c^						
Additional employment to sex work	No	1.0		1.0	1.0	
	Yes	1.50 (1.00–2.23)	0.05	1.81 (1.00–3.26)	2.26 (0.70–2.28)	0.4
Hunger – missed a meal in the last 7 days due to financial constraints	No	1.0		1.0	1.0	
	Yes	1.69 (1.15–2.47)	0.007	1.16 (0.65–2.06)	2.44 (1.37–4.35)	0.06
Any recent sexual and/or physical non-IP violence	No	1.0		1.0	1.0	
	Yes	1.96 (1.31–2.95)	0.001	1.44 (0.79–2.66)	2.84 (1.54–5.23)	0.1
Any recent sexual and/or physical IP violence	No	1.0		1.0	1.0	
	Yes	1.25 (0.82–1.91)	0.3	1.29 (0.67–2.50)	1.20 (0.62–2.33)	0.3
Recent arrest	No	1.0		1.0	1.0	
	Yes	1.24 (0.81–1.88)	0.3	1.15 (0.61–2.17)	1.32 (0.69–2.50)	0.7

a Adjusted Wald test

b Adjusted for all *a priori* potential confounders (age at baseline, religion, SES, literacy, ever married) and clinic

c Adjusted for all *a priori* potential confounders (age at baseline, religion, SES, literacy, ever married), clinic, violence from non-IPs, violence from IPs, hunger, additional employment to sex work and clients per week

When we examined for interaction with counselling, there was evidence that the association between higher literacy levels and reduced risk of suicidal behaviour was stronger amongst women who attended counselling (aOR 0.35; 95% CI 0.18–0.65; *p* 0.01) compared to women who did not attend counselling (aOR 1.14; 95% CI 0.61–2.13) (Table 6). There was some evidence that the association for the protective effect of higher SES on suicidal behaviour was stronger amongst women who attended counselling (aOR 0.33; 95% CI 0.16–0.66; *p* 0.07 *v*. those who did not attend counselling: aOR 0.54; 95% CI 0.26–1.07). Among women who attended counselling there was some evidence for a stronger association between recent hunger and suicidal behaviour (aOR 2.44; 95% CI 1.37–4.35; *p* 0.06) compared to those who did not attend counselling (aOR 1.16; 95% CI 0.65–2.06).

## Discussion

Overall there was a decline in mental health problems but persistently high levels of suicidal behaviours during the study. To our knowledge this is the first study among FSWs to examine longitudinal associations between common risk factors and mental health problems and suicidal behaviours. We found that being married, recent violence from non-IPs and food insecurity were strongly associated with mental health problems and suicidal behaviours. Older women, women of Muslim religion and women with lower client volumes were more likely to have mental health problems. Indicators of poverty and financial insecurity including low SES, low literacy levels and additional employment to sex work were associated with increased risk of suicidal behaviours. Almost half of women in our study took up counselling sessions indicating a high demand for counselling services. Attending counselling modified the risk association between literacy, SES, hunger and suicidal behaviour.

There was a decline in the prevalence of all mental health problems during the study, with approximately one in three women reporting a mental health problem at baseline and just one in ten at endline. The greatest decline was seen between baseline and midline. Results from a recent systematic review examining mental health problems among FSWs in LMICs found that the pooled prevalence for depression was 41.8% (95% CI 35.8–48.0%), anxiety 21.0% (95% CI 4.8–58.4%), PTSD 19.7% (95% CI 3.2–64.6%), recent suicidal ideation 22.8% (95% CI 13.2–36.5%) and recent suicide attempt 6.3% (95% CI 3.4–11.4%) (Beattie *et al*., 2020). At baseline, these estimates are similar to the prevalences found in our study but at endline the prevalence of mental health problems among women in our study is closer to that of the general population in Kenya (World Health Organisation, 2017). In contrast, reported suicidal behaviours remained static throughout the study. There was a high proportion of persistent suicidal behaviours and mental health problems throughout the study. Longer duration of untreated mental health symptoms is associated with worse mental health outcomes (Amati *et al*., 2018) and a history of previous suicidal thoughts/behaviours significantly increases the lifetime risk of further suicide attempts and completed suicide (McHugh *et al*., 2019; World Health Organisation, 2014). There is a need for early interventions to address mental health problems and suicide risk among FSWs.

Reasons for a decline in mental health problems are likely to be multi-faceted including increased access to counselling as well as access to services addressing syndemic risks such as alcohol/substance use counselling and violence reduction programmes. In addition to counselling, the process of participating in the study may have had some therapeutic effect. In qualitative interviews women reported the benefits of having a safe space to talk about their experiences during study visits. Evidence-based brief psychological interventions such as the HAP have been shown to be beneficial at treating moderate to severe depressive symptoms as well as having an impact on secondary outcomes including a reduction in reported IPV (Patel *et al*., 2017) among primary care populations in India. Unfortunately, formal assessment of the effect of the counselling service on mental health problems in our study was limited due to women not being randomised to counselling and most only able to attend <3 in person sessions. However, the high uptake and successful delivery of a brief counselling intervention, based on the HAP, emphasises the need for these services to be embedded within existing sex worker services. The HAP has not previously been used in sub-Saharan Africa or with sex worker populations – future research should consider formally evaluating interventions such as the HAP among FSWs.

The COVID-19 epidemic in Kenya and globally forced many women out of sex work and into alternative employment or unemployment. Between midline (May 2020) and endline (June 2020–Jan 2021) surveys, a variety of measures to curb SARS-CoV-2 transmission including curfews, closure of sex work hot spots, restriction of movement and social distancing had major impacts on the sex work industry (Kimani *et al*., 2020). If sex work and its associated risk factors were driving poor mental health then this may have had positive effects on women's mental health. For example, the prevalence of violence from clients and harmful alcohol/substance use declined from baseline to endline, which may be due to women being less exposed to these risks. However, due to sex work being criminalised and stigmatised in Kenya, women reported difficulty accessing government financial support schemes during COVID-19 (Kimani *et al*., 2020). Globally sex worker organisations have reported exclusion from emergency financial protection schemes (Iversen *et al*., 2020). This was reflected in high levels of food insecurity (recent hunger) reported by women in our study which increased from a third to over 50% at endline, with a strong association between recent hunger and mental health problems and suicidal behaviours. In addition, low SES, requiring an additional employment to sex work and lower client volumes were associated with either mental health problems or increased suicide risk, emphasising the effects of economic insecurity on mental health. Among women who attended counselling there was an increased strength of association between hunger and suicidal behaviour compared to those who did not attend counselling indicating that women may have sought counselling due to the stresses of food insecurity. A recent commentary (Kimani *et al*., 2020) on the effects of COVID-19 on sex work in Nairobi found that one of the major causes of stress for FSWs was due to food insecurity. However, they also reported that pre-existing HIV programmes and sex worker organisations in Nairobi took steps to rapidly identify and respond to their community's needs, for example, SWOP clinics re-organised their counselling services to operate by phone and sex work leaders organised the distribution of food vouchers (Kimani *et al*., 2020). This highlights the resilience and adaptability of sex worker groups and local HIV programmes. In order to avoid inequalities being further exacerbated, governments and organisations working with FSWs need to ensure that women have access to financial support during public health emergencies such as COVID-19.

To our knowledge, this is the first study among FSWs in LMICs to find a longitudinal association between recent violence from non-IPs and subsequent mental health problems and suicidal behaviours. Previous cross-sectional data including from a recent systematic review among FSWs reported a pooled unadjusted OR between recent violence and depression of 2.3 (95% CI 1.3–4.2) (Beattie *et al*., 2020), and two studies among FSWs in South Africa (Coetzee *et al*., 2018) and Kenya (Roberts *et al*., 2018) have linked recent violence and PTSD. Overall violence from any perpetrator declined but the strong temporal association between violence and mental health problems/suicidal behaviours indicates that violence remains a major risk factor for FSWs mental health and overall health and wellbeing. There is now evidence that violence interventions can be effective in reducing violence against women in LMICs (Abramsky *et al*., 2014; Kapiga *et al*., 2019) as well as among FSWs (Beattie *et al*., 2015). Future mental health and violence interventions for FSWs should consider how these issues can be addressed in parallel, for example, ensuring women reporting violence are referred for mental health support.

There was no association found between HIV status or STIs and mental health or suicidal behaviour outcomes in our study. This contrasts to findings from Beattie *et al*.'s systematic review which found an association between depression and HIV (OR 1.4; 95% CI 1.1–1.8; *n* = 4 studies) and suicidal ideation and HIV (OR 1.4; 95% CI 1.1–1.8; *n* = 2 studies), however these represent a small number of cross-sectional studies which may not be generalisable to all settings (Beattie *et al*., 2020). Possible explanations are that FSWs living with HIV benefit from access to regular support delivered through HIV/STI services at SWOP clinics including peer support and violence prevention programmes (Bhattacharjee *et al*., 2019), which may have positive impacts on their mental health. In addition, most HIV-positive women in our study have known their status for a number of years, so the initial shock of diagnosis may have diminished and there have been efforts in recent years to destigmatise HIV by peer educators discussing their own HIV status.

### Strengths and limitations

A major strength of our study is the use of validated tools to assess mental health and violence as well as the use of longitudinal data to assess the directionality of association between exposures and mental health problems. Approximately half of the estimated FSW population in Nairobi are in active follow-up at one of the seven SWOP clinics, from where we drew our sample. Due to the stigmatised nature of sex work it is possible that the most vulnerable women may have been under-represented because they were not registered at a SWOP clinic. There is potential for under-reporting of sensitive topics including mental health, alcohol/substance use and violence.

## Conclusion

The mixed findings, of a decline in mental health problems but persistently high suicidal behaviours during the study, may reflect benefits of taking part in the study such as access to counselling services as well as the more complex effects of COVID-19 on sex work, with increased financial insecurity, but reduced risk factors associated with the sex work environment (i.e. violence, alcohol use). There was high uptake and acceptability of a brief counselling service among FSWs in our study. Future research should focus on evaluating the effectiveness of psychological interventions, tailored for FSWs. This is the first longitudinal study among FSWs to find that recent violence and financial insecurity are major longitudinal risk factors for poor mental health. There is an urgent need for interventions addressing up-stream drivers of poor mental health for FSWs, such as financial support and violence prevention programmes.

## Supplementary material

For supplementary material accompanying this paper visit https://doi.org/10.1017/gmh.2022.44. click here to view supplementary material
