# Supplementary material for: Longitudinal experiences and risk factors for common mental health problems and suicidal behaviours among female sex workers in Nairobi, Kenya
Source: Glob Ment Health (Camb). 2022 Aug 18;9:401–15. doi: 10.1017/gmh.2022.44 (PMC9806968; doi:10.1017/gmh.2022.44)
Supplement: Supplementary file 1 [file S2054425122000449sup001.docx]

# Appendix A

|  |  | Completed follow up (n=877)  N (%) | Did not complete follow up (n=126)  N (%) | Pearson Chi square^2^ |
| --- | --- | --- | --- | --- |
| **Age (years)** | <25 | 182 (20.8) | 30 (23.8) |  |
|  | 25-34 | 302 (34.4) | 51 (40.5) |  |
|  | 35+ | 393 (44.8) | 45 (35.7) | 0.2 |
| **Literacy** | Illiterate | 243 (16.3) | 23 (18.3) |  |
|  | Literate | 734 (83.7) | 103 (81.8) | 0.6 |
| **Religion** | Catholic | 338 (38.5) | 37 (29.8) |  |
|  | Protestant | 461 (52.6) | 73 (58.9) |  |
|  | Muslim | 41 (4.7) | 5 (4.0) |  |
|  | Other/None | 37 (4.2) | 9 (7.3) | 0.2 |
| **Socio-economic status (SES)** | Low/low-middle | 355 (40.6) | 46 (36.5) |  |
|  | Middle | 174 (19.9) | 26 (20.6) |  |
|  | Upper middle/upper | 346 (39.5) | 54 (42.9) | 0.7 |
| **Marital status** | Ever married | 188 (21.4) | 28 (22.2) |  |
|  |  | 689 (78.6) | 98 (77.8) | 0.8 |
| **No. of children†** | None | 37 (4.4) | 3 (2.7) |  |
|  | 1-2 | 563 (67.4) | 81 (71.8) |  |
|  | 3+ | 235 (28.1) | 29 (25.7) | 0.5 |
| **Income** | Additional employment to sex work | 498 (56.8) | 73 (57.9) |  |
|  |  | 379 (43.2) | 53 (42.1) | 0.8 |
| **Hunger** | Missed a meal in the last 7 days due to financial constraints | 587 (67.0) | 83 (66.4) |  |
|  |  | 289 (33.0) | 42 (33.6) | 0.9 |
| **Place of sex work** | Lodge/hotel/rented room | 792 (91.1) | 115 (91.3) |  |
|  | Other public place | 24 (2.8) | 4 (3.2) |  |
|  | Home | 53 (6.1) | 7 (5.7) | 0.9 |
| **Client volume/week** | Median |  |  |  |
|  | <5 | 534 (61.5) | 73 (57.9) |  |
|  | 5-9 | 215 (24.8) | 35 (27.8) |  |
|  | 10+ | 119 (13.7) | 18 (14.3) | 0.7 |
| **Condom use last sex** | No | 207 (23.6) | 29 (23.2) |  |
|  | Yes | 669 (76.4) | 96 (76.8) | 0.9 |
| **HIV status** | Negative | 637 (72.6) | 109 (86.5) |  |
|  | positive | 240 (27.4) | 17 (13.5) | 0.001 |
| **Migrated for sex work** | Sex work outside Nairobi last 6 months – no | 637 (73.4) | 93 (73.8) |  |
|  | yes | 231 (26.6) | 33 (26.2) | 0.9 |
| **Any recent sexual and/or physical non-IP violence** | No | 409 (46.6) | 47 (37.3) |  |
|  | Yes | 468 (53.4) | 79 (62.7) | 0.05 |
| **Any recent sexual and/or physical IP violence** | No | 609 (69.4) | 84 (66.7) |  |
|  | yes | 268 (30.6) | 42 (33.3) | 0.5 |
| **Recent arrest** | No | 613 (69.3) | 88 (69.8) |  |
|  | yes | 264 (30.1) | 38 (30.2) | 0.9 |
| **Depression/anxiety** | No | 667 (76.2) | 91 (76.2) |  |
|  | Yes | 208 (23.8) | 35 (27.8) | 0.3 |
| **PTSD** | No | 751 (86.4) | 105 (84.0) |  |
|  | yes | 118 (13.6) | 20 (16.0) | 0.5 |
| **Recent suicidal behaviour** | No | 791 (90.2) | 111 (88.1) |  |
|  | Yes | 86 (9.8) | 15 (11.9) | 0.5 |
| **Alcohol use problem** | No | 616 (70.6) | 81 (64.3) |  |
|  | Yes | 257 (29.4) | 45 (35.7) | 0.2 |
| **Other substance use problem** | No | 601 (68.5) | 80 (63.5) |  |
|  | yes | 276 (31.5) | 46 (36.5) | 0.3 |
| **Recent STI** | No | 761 (86.8) | 106 (84.1) |  |
|  | yes | 116 (13.2) | 20 (15.9) | 0.4 |
